# Initial heritable genome editing: mapping a responsible pathway from basic research to the clinic

**DOI:** 10.1007/s11019-022-10115-x

**Published:** 2022-11-22

**Authors:** Robert Ranisch, Katharina Trettenbach, Gardar Arnason

**Affiliations:** 1 Junior Professorship for Medical Ethics with a Focus on Digitization, Faculty of Health Sciences Brandenburg, University of Potsdam, Am Mühlberg 9, 14476 Potsdam, Golm, Germany; 2 Research Unit “Ethics of Genome Editing”, Institute of Ethics and History of Medicine, University of Tübingen, Gartenstraße 47, D-72074 Tübingen, Germany; 3 University of Akureyri, Norðurslóð 2, 600 Akureyri, Iceland

**Keywords:** Heritable genome editing, Germline interventions, Research ethics, CRISPR, Translational ethics, Reproduction, First-in-human trials

## Abstract

Following the Second Summit on Human Gene Editing in Hong Kong in 2018, where the birth of two girls with germline genome editing was revealed, the need for a responsible pathway to the clinical application of human germline genome editing has been repeatedly emphasised. This paper aims to contribute to the ongoing discussion on research ethics issues in germline genome editing by exploring key issues related to the initial applications of CRISPR in reproductive medicine. Following an overview of the current discussion on bringing germline genome editing into clinical practice, we outline the specific challenges associated with such interventions and the features that distinguish them from conventional clinical testing of new medical treatments. We then review proposed ethical requirements for initial heritable genome editing, such as the absence of reasonable alternatives, the existence of sufficient and reliable preclinical data, appropriate informed consent, requirements related to safety, and long-term follow-up.

## Introduction

We have witnessed a rapid development of new genome editing tools in recent years, including CRISPR/Cas9. These and other tools, such as ZNF, TALENs, and base editors, could pave the way for the development of new somatic therapies in medicine (Porteus 2019). As the proof-of-concept has previously been demonstrated (Liang et al. 2015; Ma et al. 2017), the modification of the human embryo seems feasible (Lea and Niakan 2019; Wolf et al. 2019). While the development of somatic therapies is welcomed, so-called germline therapies or heritable genome editing (HGE) are much more ethically controversial (van Dijke et al. 2018; Almeida and Ranisch 2022).

Since November 2018, when Chinese scientist He Jiankui proclaimed the birth of two gene-edited babies (Greely 2019a), there has been an intensified and ongoing dispute on the ethics and the governance of HGE. Much debate has taken place on how applications of genome editing technologies should be regulated or whether they should be banned (Brokowski 2018; Ormond et al. 2017). As a consequence of this first-in-human application, another moratorium has been proposed, aiming at an international ban on HGE for 3 years (Lander et al. 2019).

While parts of the scientific community see the need to limit plans to integrate HGE into clinical care, the concluding statement by the organising committee of the Second Summit on Human Gene Editing (Hong Kong) adopted a different tone (Ranisch and Ehni 2020). A few days after the alleged birth of “Lulu” and “Nana”, and 3 years after the controversial first use of CRISPR on in vitro embryos (Liang et al. 2015), the organising committee concluded:

‘… the scientific understanding and technical requirements for clinical practice remain too uncertain and the risks too great to permit clinical trials of germline editing at this time. Progress over the last three years and the discussions at the current summit, however, suggest that it is time to define a rigorous, responsible translational pathway toward such trials.’ (NASEM 2019, p. 7)

The need for such a translational pathway has been emphasised repeatedly following the Hong Kong summit; ‘after the storm’, a responsible path for genome editing is needed (Daley et al. 2019). The recent report from the National Academy of Medicine, the National Academy of Sciences, and the Royal Society makes recommendations for such a translational pathway, focusing on the scientific aspects while the ethical aspects are explicitly bracketed off (National Academy of Medicine et al.  2020). The growing literature on the ethics of HGE includes numerous publications that address specific ethical issues; however, they do not offer a comprehensive review of the different ethical obstacles on the path from bench to bedside. In this article, we aim to fill this gap by reviewing the ongoing efforts to find a translational pathway for HGE. Furthermore, we aim to contribute to the current discussion on research ethics issues related to germline genome editing by exploring key issues related to the initial applications of CRISPR in clinical research.

In the next section, we provide a brief overview of the recent developments involving questions of whether and how to adapt HGE to clinical practice. This overview will set the stage for our discussion of individual ethical issues in the two sections that follow.  In those two sections we first outline the specific challenges associated with such interventions and what distinguishes them from conventional clinical testing of new medical treatments. Then we review proposed ethical requirements for initial HGE, such as the absence of reasonable alternatives, the existence of sufficient and reliable preclinical data, appropriate informed consent, requirements related to safety and benchmarks, intergenerational monitoring, and long-term follow-up studies. We consider these to be the main ethical aspects of translating HGE from the bench to the bedside, judged by a non-systematic review of the relevant literature and our knowledge of the nature of translational research on human genome editing.

## Ongoing efforts to pave the pathway to the clinics

Interventions in the human germline have thus far been seen as a red line, either because of the technical limitations of genetic modification or for ethical reasons. This has changed rapidly during the last decade (Ranisch and Ehni 2020). While something close to a moratorium was being proposed by leading scientists following the initial experiments on preimplantation embryos with CRISPR in 2015 (Baltimore et al. 2015), in 2017, the U.S. National Academies of Sciences, Engineering, and Medicine (NASEM) were already pushing research on HGE forward (Baylis 2017). In a comprehensive report published in 2017, the NASEM were the first renowned institutions to openly embrace the potential future application of HGE, outlining a set of criteria for initial clinical trials (NASEM 2017). One year later, the U.K. Nuffield Council on Bioethics (2018a) released a report on genome editing in the context of human reproduction after a general statement on genome editing had previously been published in 2016 (Nuffield Council on Bioethics 2016). Most notably, the Nuffield Council did not only give a ‘green light’ for some therapeutic applications of HGE (Drabiak 2020), but it also seemed to allow for some forms of enhancement through its focus on the ‘welfare’ rather than the health of future persons (Gyngell, Bowman-Smart, and Savulescu 2019). Furthermore, the Nuffield Council on Bioethics made a series of brief recommendations that would have an impact on future clinical trials; for example, that possible applications should be licensed on a case-by-case basis and should be closely monitored (Nuffield Council on Bioethics 2018b).

With the birth of the world’s first gene-edited babies “Lulu” and “Nana” in 2018, further efforts were made to steer future research in a responsible direction. In 2019, the National Academy of Medicine, the National Academy of Sciences in collaboration with the British Royal Society installed the International Commission on the Clinical Use of Human Germline Genome Editing. In their final 2020 report, they proposed six different categories for the use of HGE, ranked by priority, and suggested that initial clinical use should be restricted to cases of serious monogenic diseases (National Academy of Medicine et al.  2020). In addition, in 2019, the World Health Organisation (WHO) established a working group to investigate the ethics and governance of translational genome editing and launched the Human Gene Editing Registry. This registry includes clinical trials of somatic genome editing, but it will also cover research other than clinical trials involving human embryos and germline cells in the future. The working group published two reports in 2021 on human genome editing that focused on questions related to the governance of HGE and made recommendations for individual jurisdictions (WHO 2021a, WHO 2021b). In their documents, the working group made recommendations and included points to consider regarding the potential licensing of both somatic and HGE, local stakeholder engagement, and the implementation and further development of a global registry for clinical research in genome editing. Addressing the requirements for a responsible translational pathway for HGE has begun. Various authors and institutions have stressed the need to develop ethically sound approaches to bring HGE to the clinic (Cwik 2017). Some authors have started to outline the requirements for such a pathway (Cwik 2020b; Brokowski and Adli 2020; Jonlin 2020; Daley et al. 2019; Savulescu and Singer 2019), and a more detailed roadmap was proposed by Evitt et al. (2015).

Throughout these developments, various ethical issues have arisen, and suggestions have been made for how to manage these issues to find a responsible way to bring human genome editing into the clinic. In the remaining sections, we will discuss these issues in more detail and depth.

## The peculiar nature of initial HGE experiments

Considering the history of artificial reproductive technologies (ARTs), developments such as intracytoplasmic sperm injection (ICSI) were adapted directly from the lab to clinical use and eventually became routine applications, often with limited knowledge of the effectiveness and safety of these innovations (Harper et al. 2012). Any future attempt to move HGE from bench to bedside will be unlike the clinical testing of new medical treatments. However, before becoming clinical routine, some form of licensing will be necessary, and this should be accompanied by additional precautions. We will use the term ‘initial heritable genome editing (i-HGE)’ to refer to the first experiments and studies that would precede a possible introduction of HGE into routine clinical practice.

Since the second half of the 20th century, considerable efforts have been made in the formulation of rules and guidelines for responsible experimentation involving humans. Even though central documents on research ethics, such as the Declaration of Helsinki (WMA 2013), do not address clinical research that deliberately targets the germline, core principles of human research ethics are still applicable in this field. For example, clinical research requires approval from a research ethics committee, informed consent from the participant must be obtained, and risks associated with the experiment should be minimised to a reasonable level. The Declaration of Helsinki allows for unproven interventions in the treatment of an individual patient in the absence of alternatives (WMA 2013, principle 37), and the genome editing that led to the birth of “Lulu” and “Nana” may be considered such a case. However, as the Declaration of Helsinki maintains, an unproven intervention may only be justified if ‘it offers hope of saving life, re-establishing health or alleviating suffering’ and if the intervention is to ‘subsequently be made the object of research’ (WMA 2013, principle 37). Given the risks, uncertainties, and the clear experimental nature of current human genome editing, He Jiankui’s experiment was not a step towards a responsible pathway for i-HGE. Circumventing the ethical requirements of clinical research by conducting high-risk experiments under the guise of ‘unproven interventions’ is highly unethical (Arnason 2019; Lyerly et al. 2001).

Responsible i-HGE must be conducted in the context of clinical research; however, unlike most clinical research, we find an extended circle of subjects for i-HGE. An experiment does not only involve the mother-to-be, but also the future child. While this is also the case for some other trials (e.g., foetal therapy), i-HGE is exceptional; some of the research subjects and individuals affected by the study do not exist prior to the experiment, including the future child and other individuals further down the germline (i.e., subsequent generations). This raises two questions: what specific challenges arise from the peculiar nature of i-HGE, and can i-HGE be considered a form of therapy, specifically regarding the potential benefits for the offspring?

### The translational dilemma

As for first-in-human trials, finding a responsible pathway for research with HGE leads to a dilemma, which we will refer to as the ‘translational dilemma’ (Ranisch 2020b). On the one hand, i-HGE could not be justified before it is proven to be sufficiently safe; on the other hand, in vivo research is necessary to provide evidence for the safety of such experiments. Considering the uncertainties, i-HGE seems irresponsible, which eo ipso prevents us from moving forward in the field. It follows from this dilemma either that i-HGE may be too high-risk to be conducted altogether (Lanphier et al. 2015) or that some risks should be accepted to gain further knowledge. As Poulton and Oakeshott maintained in a similar context, namely for mitochondrial replacement therapy (MRT): ‘The only way to find out about safety is to license the first treatment. … The risk of generating abnormal babies is unknowable until such a trial is done’ (Poulton and Oakeshott 2012).

The translational dilemma is not a unique characteristic of i-HGE but also occurs in other clinical research. However, in the case of i-HGE, the stakes are higher. Unlike the future offspring in i-HGE experiments, potential subjects in a research study can provide consent for participation. In principle, they can and must be adequately informed about the purpose and potential risks of the study. Then, competent subjects can decide for themselves whether they are willing to accept the associated risks. However, this is not always possible. Research sometimes involves subjects that are incapable of providing informed consent, such as people with advanced dementia, young children and infants, and—in the case of clinical research on foetal therapies—unborn babies. As the Declaration of Helsinki states, such individuals ‘must not be included in a research study that has no likelihood of benefit for them unless it is intended to promote the health of the group represented by the potential subject’, and ‘the research entails only minimal risk and minimal burden’ (WMA 2013, principle 28).

### Is HGE a form of therapy?

To assess the potential benefits of any HGE (and i-HGE in particular), it should be noted that the status of such interventions is not uncontroversial. Regarding HGE, there are three main positions. (i) It could be considered as some form of therapy or prevention because, if successful, it prevents a future person from developing a specific disease by ‘treating’ an embryo, from which this individual develops. In this context, HGE and similar technologies are sometimes deemed ‘embryo-therapy’ (Wrigley et al. 2015) or ‘pre-emptively therapeutic’ (Cavaliere 2018). It has also been suggested that HGE could then be ‘life-saving’ for embryos if it cures a condition that is lethal in the neonatal period (Savulescu and Singer 2019). According to another perspective, (ii) HGE is more akin to a public health intervention because it does not cure an existing patient but instead reduces the prevalence of a specific disease, morbidity, or mortality (Schaefer 2020). In contrast, (iii) it could also be argued that HGE is essentially a form of fertility or reproductive treatment as it does not cure a manifest disease in a patient but rather increases the chance that a couple has healthy, genetically related offspring (Rulli 2019).

The way HGE is perceived is likely to affect the evaluation of future research with i-HGE. The closer HGE comes to being perceived as a form of treatment or therapy aimed at the offspring, the more likely it is that associated risks for offspring could be justified, as the primary aim is then to improve their (future) health and welfare. If HGE is seen through the lens of public health or as an extended fertility treatment, i-HGE may not be justified because it puts the health or welfare of the offspring unjustly at risk for the benefit of other parties (e.g., society, parents). The legitimacy of i-HGE depends on the aim of benefitting the offspring and consequently on what those potential benefits for the offspring are and whether they outweigh the risks.

### HGE and the benefits for the child

If we assume the likelihood of benefit to be a condition for ethically justifiable research with subjects incapable of providing informed consent, as we suggest above, it is unclear whether this condition can be fully met in the case of i-HGE. While the specific health-related risks associated with i-HGE are the subject of the section "Safety criteria and benchmarks" below, here we will focus on the principal question of whether such interventions might benefit (or harm) the created child.

Comparing i-HGE to conventional clinical research with patients (or even human foetuses) reveals a striking difference. Subjects enrolled in a typical clinical trial are always exposed to the associated risks of the study; however, they could potentially profit from experimental therapies. The same is true for a foetus that could have an increased chance of survival due to the experimental treatment. In the case of i-HGE, the situation is different. As in conventional clinical research, the woman bears risks (and could also benefit from a successful experiment). However, at the same time, such experiments can lead to the existence of another person (i.e., the offspring). Unlike conventional experiments, in i-HGE, the conception of a ‘subject’ is part of the research. Therefore, such interventions are identity-affecting—they are a necessary condition for the existence of an identifiable individual. For the very reason that this individual did not exist prior to the intervention, it is more difficult to maintain that this person could (even potentially) benefit from or be harmed by such an experiment.

It is a contested issue whether an event or action (e.g., an i-HGE experiment) that causes a person to exist can harm or benefit this person or whether such an event is neutral for this specific person (Sparrow 2021). While some argue that identity-affecting actions can neither harm nor benefit future people, others consider it possible (for discussion, see Roberts and Wasserman 2009; Ranisch 2021, p. 231–255). Some claim that identify-affecting actions can harm a future person, without maintaining that these actions can equally benefit this person. There seems to be a familiar common sense view that the possibility of a future person’s life being very bad provides a reason not to bring this person into existence as doing so would constitute some form of harm. In contrast, the possibility that a future person’s life is wonderful or healthy does not seem to provide a corresponding reason to bring this person into existence (for discussion, see McMahan 1981), as doing so would not in itself benefit that person. In the case of i-HGE, it could thus be maintained that such experiments may only cause harm for the offspring (e.g., in the case of failure), but provide no corresponding benefit (in the case of success). In the following, we will not resolve the question of whether identity-affecting actions can harm and/or benefit the future person but will acknowledge different views equally.

Consider a case where a couple are known carriers of a serious genetic disease but have the strong desire to have a genetically related, healthy child (see Table 1). If the couple conceives naturally (A), it is highly likely that their child will be severely affected by the genetic condition. This would certainly frustrate the desire for healthy progeny and— depending on the view—could be seen as a form of harm to that child. If the intended mother participates in an i-HGE experiment, it could give the couple the chance to have a healthy child if the experiment succeeds (B.i.); however, it also carries the risk of being ineffective or having harmful side effects for the child (B.ii.). Again, it depends on the respective point of view whether this is seen as a benefit (B.i.) or as a harm (B.ii.) to the child or whether any outcome is considered neutral towards the offspring. Finally, the couple could give up their desire to have a genetically related child and refrain from procreation (C), which would be frustrating but would have no effect, good or bad, on the non-existing child.

**Table 1:** Options for prospective parents who are carriers of serious genetic diseases and possible outcomes

	Outcome for the (intended) parents	Outcome for the offspring
A) conceiving naturally	frustration	harm or neutral
B.i.) i-HGE (successful)	benefit	benefit or neutral
B.ii.) i-HGE (unsuccessful)	frustration	harm or neutral
C) refraining from procreation	frustration	neutral

What becomes evident in this case is that i-HGE and HGE in general could clearly be beneficial to the parents by serving their interest of having a healthy child. For that reason, it is sometimes argued that the parental desire for genetically related and healthy offspring is the driving force and main justification behind i-HGE (Andorno et al. 2020; Malmqvist 2021). Moreover, it is certain that refraining from procreation (and refraining from i-HGE) will not harm any child because they would never exist. However, it remains an open question whether any HGE could benefit (in case of success) or harm (in case of failure) the future person (Ranisch 2020a), or whether it is neutral to the future person created (this is why the outcome for offspring is indicated as possibly neutral in each case in Table 1). If the possibility of harm by creating is granted, it remains to be seen whether the desired benefits for the intended parents could outweigh the risks for the child. In this regard, there are three plausible positions. It could be maintained that:

I. HGE is inherently non-beneficial for the child but could convey risks and thus cannot be justified;
II. HGE is inherently non-beneficial for the child but could be beneficial for the parents and could therefore be justified;
III. some HGE could be beneficial for both the child and the parents and could therefore be justified.

Only proponents of the second or third position may consider i-HGE to be permissible in principle. However, even for these, it should be clear that, in view of the uncertain benefits for the offspring or the subsequent possibilities to avoid harm to offspring (by refraining from reproduction), higher safety requirements would have to apply to corresponding experiments. It remains unclear how a high level of safety of i-HGE can be established without conducting i-HGE experiments; therefore, the translational dilemma has not been resolved.

## Mapping a responsible pathway

There are several obstacles to establishing the safety and efficacy of i-HGE and finding a responsible pathway. Along the path to potential clinical use, we will review some points for consideration from the current literature. These include safer alternatives, the difficulty of producing reliable preclinical data, the problem of recruiting research subjects, questions about autonomy and consent, challenges in establishing safety criteria and benchmarks, and questions about the extent to which long-term follow-up is required and feasible.

### Alternatives

Because of the high-risk nature of germline modifications, HGE is widely considered by some as the last resort for couples wishing to have genetically related healthy children. As stated by the NASEM, i-HGE should be permitted only in the absence of reasonable alternatives (NASEM 2017). However, in most cases, preimplantation genetic testing (PGT) and embryo selection is an alternative to preventing the transmission of diseases. Only in very few cases (e.g., when both parents are homozygous for an autosomal recessive disease) would HGE be the only option to prevent the transmission of a monogenetic disease (Ranisch 2020a). In addition, applications are conceivable in which HGE, although not without alternatives, could increase the chances of having a healthy child (e.g., if the number of available oocytes or unaffected embryos is so low that editing of otherwise discarded embryos is an option) (Steffann et al. 2018).

Two aspects need to be considered. First, beyond the possibility of refraining from procreation, we must question whether adoption or third-party egg or sperm donation can be regarded as a reasonable alternative to HGE. The continuing demand for ARTs when adoption is available is an indication of the value placed on having genetically related children. There are numerous reasons why people may desire to have a genetically related child, including a desire for a physical resemblance between the parent(s) and child, family resemblance, to continue one’s life in some sense through the child, or for the genetic relationship itself (Rulli 2016). While i-HGE imposes risks on future persons and demands on society, rejecting adoption or third-party egg or sperm donation as reasonable or preferable alternatives to i-HGE requires a justification for the moral value of a genetic relationship between parents and offspring; however, this debate is beyond the scope of this article (for an overview of this issue, see Segers et al. 2019).

Second, alternatives should be considered not only regarding ARTs, but also regarding common therapeutic or preventive options. For example, it was argued that the experiments by He Jiankui targeting *CCR5* met a medical need as ‘there is no cure or vaccine for HIV’ (Cohen 2018). This argument fails to recognise that conventional alternatives to prevent and treat HIV infection are available, efficient, and are associated with lower risks than i-HGE. Before conducting first clinical experiments with HGE, other medical options should always be considered, including the development of future somatic gene therapies.

Although i-HGE may only be justifiable if there are no reasonable alternatives, it should be noted that the lack of alternatives could also increase the vulnerability of the prospective parents and thereby produce new moral concerns (Malmqvist 2021). If a couple expresses a strong desire to have genetically related offspring that could only be fulfilled by participation in an i-HGE experiment, this option may be impossible to reject.

### Reliable preclinical data

Even if proof of safety cannot be achieved without initial trials, it is necessary to have reliable preclinical data before any i-HGE can be conducted. Prior to calls for a responsible pathway in the wake of the birth of “Lulu” and “Nana”, commentators such as Evitt et al. (2015, p. 26) had previously highlighted the necessity for ‘proof of concept [for HGE] by applying putative therapeutic gene edits to relevant somatic cells and multigenerational animal models’. More recently, the European Society of Human Genetics and the European Society of Human Reproduction and Embryology stressed the significance of animal studies and the importance of human embryo studies (de Wert et al. 2018). Robust preclinical data is not only necessary to facilitate progression from one stage of translational research to the next; eventually, it enables competent assessment of the risks and benefits of a first-in human trial (e.g., in Phase-1 trials). However, researchers have found existing preclinical data lacking in methodological quality, which makes it difficult or nearly impossible for review boards to make competent judgements about the risks and benefits of a proposed trial.

A recent review of over 700 preclinical efficacy studies in over 100 investigator brochures addressed to institutional review boards (IRBs) at German university medical centres illustrates this point. The study found that most preclinical efficacy studies lacked sample size calculation, randomisation, and blinded outcome assessment (Wieschowski et al. 2018). While the reviewed investigator brochures were not limited to any specific clinical specialty, area of research, or i-HGE, the study highlights a wider problem in the current practice of translational clinical research, namely how poor methodological quality can jeopardise scientific validity. Moreover, further problems abound: in his 2010 book on first in-human-trials, Jonathan Kimmelman (2010) demonstrates how publication bias, conflicts of interests, and optimism bias all skew the credibility of preclinical data.

In 2013, Henderson, Kimmelman, and colleagues proposed credibility alongside internal, construct, and external validity as components of a four-part framework to minimise uncertainty between different steps in translational research (Henderson et al. 2013). Baylis and McLeod (2017) have since applied this framework to approved clinical trials of somatic genome editing, and Nordgren (2019) suggested applying this framework to translational research on HGE to observe potential uncertainties (e.g., between animal models and human embryo research).

What becomes obvious, even at a cursory glance at the quality of preclinical data in biomedicine, is that the translational path for i-HGE cannot be designed or even conceptualised without taking into account known systemic challenges and problems in the current biomedical innovations landscape.

### Recruitment and targets for i-HGE

Any plans to conduct i-HGE raise the question of how to select the first couple(s) to be recruited and what targets of interventions of the germline could justify the risks associated with such experiments. This question can be addressed from various perspectives. First, it should be determined for what purpose genome editing technologies should be used when i-HGE is planned to prevent the onset of heritable conditions or even to target genes coding for traits not associated with disease (i.e., enhancement). Even when deciding on targeting disease-associated genes, it should be determined which specific conditions would warrant initial use of HGE.

In their 2020 Report, the International Commission on the Clinical Use of Human Germline Genome Editing concluded that i-HGE should be limited to serious monogenic diseases and to ‘changing a pathogenic genetic variant … to a sequence that is common in the relevant population and that is known not to be disease-causing’ (National Academy of Medicine et al.  2020, p. 3 [recommendation 4.2]). Erika Kleiderman and colleagues likewise stated that the ‘seriousness’ of a condition is relevant to decision-making regarding the use of i-HGE, as a greater benefit could offset the risks associated with a previously untested procedure (Kleiderman, Ravitsky, and Knoppers 2019; 2020). The authors further derive the significance of seriousness from a human rights-based framework, specifically in the ‘right to science’ and the ‘right to the highest attainable health’, to bridge the divide between objective and subjective definitions of health (Kleiderman, Ravitsky, and Knoppers 2019; 2020).

Their article received varying replies, and not all respondents agreed regarding the relevance of seriousness, resulting in some debate about whether consideration should be given to seriousness regarding i-HGE. For example, author Iñigo De Miguel Beriain dismisses the significance of seriousness altogether; to him, the ‘irrelevance of the seriousness factor’ is steeped in the difference between PGT and i-HGE, the former being a mechanism of selection and the latter being ‘clearly therapeutic’ (we discuss above whether i-HGE is a form of therapy). In addition, according to De Miguel Beriain, PGT constitutes a viable alternative to i-HGE for disease prevention; in contrast, regarding the ‘right to the highest attainable health’, the true potential of i-HGE lies outside of serious conditions and expressly includes applications that could be deemed enhancement rather than therapy (De Miguel Beriain 2020).

In response to their article, Satvir Kalsi agreed with Kleiderman, Ravitsky, and Knoppers about the relevance of the ‘serious factor’ but noted that concepts such as the objectivist view of health and the ‘right to science’ remain poorly fleshed out and need to be more elaborately defined to facilitate a fruitful debate about what makes a condition ‘serious’ (Kalsi 2020). This observation highlights the elephant in the room with which the whole discourse about seriousness in i-HGE grapples: regardless of whether one considers seriousness relevant, a viable set of criteria and a working definition of what makes a condition serious are needed to discuss potential applications of i-HGE.

**Fig. 1:**
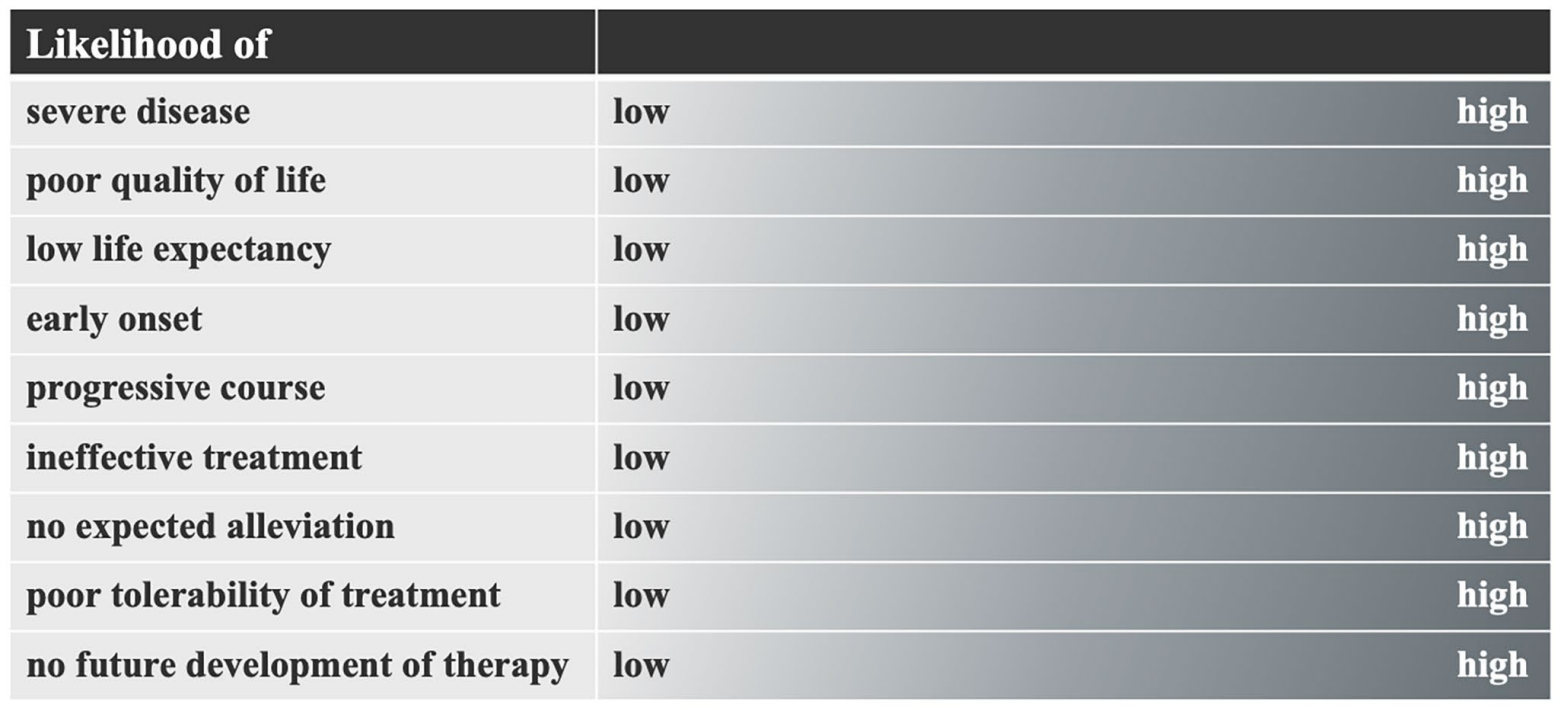
Criteria to determine the “seriousness” of a condition. (adapted from SAMS 2020)

We suggest a multilayer framework that builds upon a proposal by the Swiss Academy of Medical Sciences (SAMS) and has been brought forward in the context of PGT/prenatal testing (Fig. 1). When discussing the permissibility of i-HGE, these criteria can provide guidance to determine the permissibility depending on the prevalence and intensity of individual criteria, which together can define the seriousness of a genetic condition.

Furthermore, this framework explains why some *CCR5* mutations that confer resistance to HIV would not qualify for i-HGE. While AIDS is considered a severe disease, there are several treatment options available to alleviate and suppress the effects of the immune deficiency syndrome. Where antiretroviral therapy is available, the life expectancy of HIV-positive adults is close to the life expectancy of adults without HIV infection (Marcus et al. 2020). In addition, there is a fair likelihood that future (gene) therapies could provide a cure for HIV. Arguably, the same could be maintained for commonly discussed conditions, such as genetic deafness (Padden and Humphries 2020). As suggested by Savulescu and Singer (2019), i-HGE could instead be reserved for cases of severe disease that are lethal in the neonatal period. However, it should be noted that applying i-HGE for the prevention of a serious disease would pose a greater risk to the future child should the procedure fail in any way (for a discussion of the risks associated with i-HGE,see the section "Safety criteria and benchmarks" below).

### Consent

Informed consent is one of the main requirements in all ethical guidelines for biomedical research. In the context of i-HGE, the requirement for consent applies to the prospective parents, the gene-edited offspring, and possibly to their descendants. In this section, we will first briefly consider whether it is a problem for i-HGE that the future offspring cannot consent to the experimental genome editing procedure. Then, we will discuss the ethical issues surrounding the consent of the prospective parents.

It is the future person who will carry the burden of the adverse consequences of i-HGE; however, that person is unable to consent to the intervention to the genome. As a rule, no one can be subjected to an experiment without their informed consent. On the surface, this may seem to be a problem for, or an argument against, i-HGE (Dickenson 2020; Smolenski 2015). This objection has an intuitive appeal; however, it has been widely criticised (e.g., Harris 2016; Gyngell et al. 2017). In her book *Altered Inheritance*, Baylis (2019, p. 108) calls this criticism a red herring, noting that ‘embryos do not consent to anything.’ Prospective parents make many decisions before and during pregnancy that affect their future offspring (Ranisch 2017).

Furthermore, one can argue that the consent of the future offspring is not needed as parents are often allowed to provide consent for medical procedures on behalf of their children (Evitt et al. 2015, p. 27). According to Smolenski (2015), parents’ proxy consent for gene editing their unborn offspring is not analogous to parents consenting on behalf of their young children because, in the context of i-HGE, proxy consent would be entirely based on speculations about the child’s preferences and wishes. Nonetheless, in the case of foetal surgery, the parents do not know their offspring and yet the prospective mother ‘has the ethical and legal authority to give informed consent’ (NASEM 2017, p. 108). According to the Declaration of Helsinki, subjects that are incapable of giving consent may only be involved in research ‘if the physical or mental condition that prevents giving informed consent is a necessary characteristic of the research group’ (WMA 2013, principle 30). For example, this is true for some patients with advanced dementia but could also be extended to future persons. Therefore, i-HGE research may be justified without consent, at least if it is perceived as beneficial to the future child (Gyngell et al. 2017) (for a discussion of the benefit for the child, see section "HGE and the benefits for the child" above).

Although there is disagreement about whether the embryo’s non-consent is an issue for i-HGE, it is generally accepted that the consent of the intended mother, and arguably the consent of both prospective parents, is absolutely essential. One aspect of i-HGE that makes informed consent difficult is the possibility of adverse effects occurring either later in the life of first-generation gene-edited persons or only in subsequent generations (Rubeis and Steger 2018). This temporal and possibly generational spread of risk may require a higher standard of informed consent for i-HGE research that results in gene-edited humans coming into existence (Evitt et al. 2015).

Although the offspring carries most of the risk, the risks to and the pressures on the intended mother should not be ignored. There are risks involved with in vitro fertilisation (IVF), a procedure that the woman might choose even without i-HGE. Furthermore, there may be coercive social pressures on the intended mother of which she may not be aware (Farrell et al. 2019, p. 1074).

The voluntary participation of the prospective parents may be compromised through a strong incentive to participate in an i-HGE study if it is their only hope of having a genetically related child (without a specific genetic disease). This incentive may even be stronger when a previous pregnancy has been lost. A strong desire to have a potentially healthy child might result in psychological pressures that compromise voluntariness, as almost any risk or burden will seem acceptable or ‘worth a try’.

The consent of the prospective parents may also be compromised through a misunderstanding of the nature of the research, whether through ‘therapeutic misconception’ or ‘therapeutic misestimation’ (Horng and Grady 2003). For example, it would be a therapeutic misconception if the prospective parents have been through a number of unsuccessful infertility treatments and expect that, in the context of an i-HGE study, their infertility problem will be recognised and treated, when this is not the aim of the research. Insofar as a distinction can be made at all in the case of i-HGE between research and therapy, the primary aim is to assess the safety and effectiveness of the procedure, and the therapy itself is only a secondary aim. The misconception in this case also concerns the aim of the therapeutic aspect (infertility or a genetic disease). Therapeutic misestimation may consist of overestimating the likelihood of therapeutic success and benefit or ignorance or underestimation of experimental risk. A case has been made to avoid the term ‘therapy’ in clinical research involving foetuses to avoid such misconceptions (Deprest et al. 2011). Similarly, the phrase ‘germline therapy’ may foster a therapeutic misconception and should perhaps be eschewed.

Finally, there is the danger of viewing informed consent as the solution to all ethical problems, even though obtaining informed consent does not absolve clinicians of their responsibility towards the future child, the prospective parents, and even society (Dondorp and de Wert 2011, p. 1607). Nor does informed consent suffice to make research on humans ethical, just as its absence does not necessarily make research unethical (Emanuel et al. 2000).

### Safety criteria and benchmarks

In addition to consent to participate in i-HGE experiments, the safety of appropriate interventions for the offspring and the intended mother remains a critical factor. This requirement is in line with the medical ethical principles of nonmaleficence and beneficence. While the safety of i-HGE is one of the common demands, it often remains unclear which factors of uncertainty are associated with i-HGE and what can be evaluated as a benchmark for safety. After discussing safety aspects for the affected offspring of i-HGE, the focus is shifted to the intended mother, as she is also exposed to the risks of the experimental intervention.

Regarding the risks associated with i-HGE, a distinction can be drawn between known unknowns and unknown unknowns. When discussing genome editing platforms, known unknowns (i.e., ‘known risks’) are concerns about the precision and efficacy of a given platform. For the CRISPR/Cas-system, often discussed and well researched known unknowns include off-target and on-target effects. Off-target effects include the introduction of unintended changes to DNA at different sites in the genome than the target site, while on-target effects refer to unintended changes at the target site, such as unwanted mutations, both insertions and deletions (‘indels’), and even more complex genotypes (Lee and Kim 2018; Kosicki et al. 2018). These types of risks become apparent early on in the use of the technology and even in a basic research setting. As these can prove a technical limitation to the applicability regardless of purpose, clinical or otherwise, the known unknowns are subject to basic research themselves.

Making genome editing systems increasingly more precise is an ongoing effort, one that recently yielded systems such as prime editing that promise higher precision by circumventing the introduction of double-strand breaks (DSBs) and the subsequent error rate of the DSB repair mechanisms while enabling a greater range of possible edits than base editing (Kantor et al. 2020). Therefore, the question of the known unknowns is (to some extent) a question of the current state of advancement of the technology in question.

Unknown unknowns, as the term suggests, are much more difficult to identify and consequently more difficult to anticipate (if at all). For i-HGE, this type of risk includes adverse effects that are theoretically conceivable but would manifest only after a prolonged period. This would make it difficult to counter or even undo their effects. Here, a look at earlier technologies can inform debates on present ones. For example, research into the long-term effects of ICSI, an earlier ART, is still ongoing. Its effect on birthweight is being investigated in addition to potential increases in the prevalence of premature births or imprinting disorders (Steel and Sutcliffe 2009; Vermeiden and Bernardus 2013). Such concerns focus on the manipulation of cells in vitro and describe long-term effects from cellular reactions to techniques, such as microinjection, or even the exact composition of cell culture media (Steel and Sutcliffe 2009). These can trigger biochemical reactions, the long-term outcomes of which are much less foreseeable than estimating the likelihood of off-target effects from previous data and will show only in follow-up studies on a systematic, non-individual level.

If these and similar concerns have existed around ICSI, they are likely to exist around i-HGE. Such experiments require culturing cells in vitro and a mode of delivery of the editing platform into the cell. This necessitates an artificial crossing through the cellular membrane, for example via microinjection, electroporation, or viral vectors. Notably, these procedures raise questions of efficacy as much as the genome editing platforms themselves and are accompanied by an additional host of risks and uncertainties.

For now, the search for criteria of sufficient safety will focus on safety issues associated with the known risks of i-HGE. Previously, these have been identified as concerns surrounding the precision of HGE. Therefore, the question of safety can be rephrased again here to mean: when can a germline genome editing system be considered precise enough? The translational dilemma raised the question of when i-HGE could be considered ‘reasonably safe’ to use (i.e., ‘safe enough’). This section will outline a range of possible benchmarks for the outcomes of i-HGE. It is possible to discern between two types of benchmarks: the first type denotes standards for the precision of i-HGE, while the second type relates to the outcome of the intervention, especially regarding the welfare of the future person.

Regarding precision, multiple scenarios are conceivable. We could imagine a genome editing system that only introduces intended changes to the DNA and only at the intended sites, one that operates entirely without off-target or on-target effects (I). However, a complete absence of mutations is not a situation normally found in living cells, embryonic or somatic. Mutations are occurring constantly, both in somatic and germline cells (Scally 2016). As Greely highlighted, the germline genome of every baby has been changed in some way (Greely 2019b, p. 256). Recently, even aneuploidy and mosaicism were found to be frequent occurrences in early-stage human embryos (Starostik, Sosina, and McCoy 2020). There currently exists a natural mutation rate that is and must be tolerated in reproduction as molecular biology cannot circumvent the occurrence of mutations entirely (Schleidgen et al. 2020).

A genome editing system could introduce unwanted changes alongside intended ones to various extents. For example, a given genome editing platform could reach a precision equal to the natural mutation rate (II). Naturally, a system that ranges anywhere between (I) and (II) in its editing accuracy is possible, as is a system of an even lower accuracy than the natural mutation rate. Such a system would introduce a host of unintended changes at a rate exceeding the natural mutation rate while continuing to modify the target site as intended (III). Likewise, an editing system could fail to make the intended edit, either resulting in on-target effects, such as indels, or a failure to edit the target site altogether (IV), with or without the simultaneous occurrence of off-target edits (V).

We could consider a case where germline cells are edited to prevent a hereditary disease from being passed on to offspring. Depending on the precision of the edit, this could result in a series of outcomes for the person born following the edit regarding their quality of life:

1. The edit effectively prevents the condition. This results in a higher quality of life than is assumed for a life with the condition.
2. The edit leaves an individual in a state not worse than that with the disease.
3. The edit results in a quality of life ranging between (1) and (2).

However, in the worst case, a failed modification can put the offspring in a worse state than they would be in without the modifications and with the disease-associated mutation:

4. The edit could result in a quality of life worse than a life with the disease it initially aimed to prevent.
5. An extreme variant of an adverse outcome would be a ‘wrongful life’ defined by a quality of life so low as to not be worth living.

What remains to be seen is which level of precision would be minimally required to achieve a favourable benchmark regarding quality of life following i-HGE, such as (1). While all the proposed benchmarks are theoretically conceivable, not all of them will be equally likely, reasonable, or even applicable. While it has been proposed that the risks of i-HGE could not be ‘worse than the fate of the unedited embryos’ (Savulescu and Singer 2019), refraining from the trial or the act of reproduction would spare an embryo and future person this fate.

As the focus of the current debate has been on the safety of the child and its potential descendants, it has largely been ignored how i-HGE can also pose risks to the intended mother as she is the primary and first research subject exposed to the burdens of the experiments (Simonstein 2019; Farrell et al. 2019; Farrell, Malek, and Scott 2021). It has also been noted in the literature that there are few safeguards to protect women. As Farrell et al. (2019, p. 1072) stated, ‘virtually no discussion has taken place about establishing safeguards to ensure that female subjects are not exposed to disproportionate risk in exchange for benefits they might expect for future offspring.’

While safeguarding mechanisms for health and safety in i-HGE have been absent in general, the question has been raised whether a trial should include a mechanism for ‘termination by design’ should the experiment fail (Ishii and De Miguel Beriain 2019), e.g., if it becomes clear that the editing has not taken place as expected or if any other complications arise during the trial. For example, in the document believed to be the consent form for He Jiankui’s experiments with HGE, it was assumed that, in the case of genetic defects in the unborn child, the intended parents ‘could start the second cycle after the abortion.’^1^ While the experiment leading to the births of “Lulu” and “Nana” has since been cited as an exemplary case of flawed research ethics, it raises the important question of how to proceed when i-HGE is discovered to have failed. Historically, the question is not a new one: in the case of Louise Brown, the first child born via IVF, there is evidence that leading clinician and scientist Steptoe ‘asked for an assurance from all patients undergoing IVF and embryo transfer that they would permit an amniocentesis on any pregnancy and would agree to a termination if an abnormality was found’ (Johnson and Elder 2015, p. 42). While the question of proceedings in case of complications may not be a new one or unique to i-HGE, it should still be given consideration when designing future experiments. However, given the value of reproductive freedom and the protection of women’s bodily integrity, an obligation to terminate a pregnancy(as well as an obligation to carry the pregnancy to term) in the event of an unfavourable outcome, can hardly be an ethically justifiable option.

### Long-term follow-up

As has already been noted, potential adverse effects of i-HGE may only develop late in the offspring’s life or in subsequent generations. For this reason, long-term follow-up and health monitoring has been suggested for the offspring and following generations (NASEM 2017; Nuffield Council on Bioethics 2018a, p. 140 [para 4.95], p. 162 [recommendation 15]). While the important questions regarding long-term monitoring may only arise decades from now, infrastructure would need to be put in place simultaneously with the implementation of i-HGE to ensure effective monitoring in the future. However, there are reasons to be pessimistic about the compliance of the subjects, as previous experience from mitochondrial replacement studies and other ART research is not encouraging (Ishi 2019; Dupras-Leduc et al. 2018). In these studies, long-term follow-up was recommended or required (Department of Health 2014). In some cases, parents refused to consent to health monitoring for their child or withdrew their consent. Others were lost to follow-up due to relocation (Ishi 2019). It is likely that the interest and incentive to participate in long-term health monitoring, especially when the offspring appear healthy, will fade relatively fast. This applies to the parents, as well as the offspring and following generations when they become old enough to give or withhold consent. There is the possibility of making long-term health monitoring a legal requirement; however, this would conflict with the person’s right to autonomy and privacy (Ishi 2019; Cwik 2017).

To conduct long-term follow-up research, it will be necessary to identify and contact the offspring and possibly their future children and grandchildren. Even if they do not consent to participating in intergenerational health monitoring, there may be reasons to contact them if adverse health effects are discovered that might affect them. In this case, they would have to be traceable, which means that identifiable genetic information would be kept, possibly for decades (Cwik 2017), raising questions regarding (genetic) privacy. A further complication arises when the descendants of gene-edited offspring are not aware of their status. Being contacted and informed about the genome editing may lead to a significant psychological burden.

Even for the offspring and descendants who consent to participating in intergenerational health monitoring, such measures may represent a significant burden. Monitoring may require both time and money, especially if it involves travel to an institution where the monitoring takes place. These institutions are likely to be highly specialised and may therefore be geographically distant from the participants. The resulting financial burden for participants may require compensation. The examination itself may involve taking blood samples and a physical examination (Cwik 2017, p. 1912). In one mitochondrial replacement study, ‘whole blood and tissue samples, including buccal swabs, skin tissues, hair follicles and urine precipitates, were provided for testing’ (Ishi 2019, p. 324). Furthermore, monitoring may be extended to mental health and social issues (Thompson 2019).

Follow-up examinations would likely focus on general health, specific health issues that may have arisen in relation to i-HGE for other persons or in other experiments, and the participant’s genome. In the last case, there may be a considerable interest in whole-genome analysis of the offspring and descendants to confirm that the genome editing was successful and to identify off-target mutations and irregularities. Genomic analysis of this kind in children is problematic, both when it discovers genetic mutations due to the genome editing process and in the case of incidental findings that are relevant to the future health of the child. There are issues here regarding what should be done with such information, how to inform the parents and whether, when, and how to inform the child.

There is a question regarding the obligations of the researchers with regard to the child and their descendants. Cwik (2020a) has argued that the researchers carry the responsibility of risks and adverse effects in the child and descendants, even in case where they withdraw their consent for participating in intergenerational health monitoring. In regular clinical research, participants are recruited and can withdraw consent and end their participation at any time. In this case, the relationship between the researchers and the subject is terminated and the researchers often do not have any further obligations to the subject. However, in an i-HGE study, the researchers ‘have generated risks for the edited subjects and their descendants’ (Cwik 2020a, pp. 185–186). The researchers will therefore have some responsibility for certain aspects of the subject’s health, even if they withdraw from long-term monitoring.

Finally, the obligations of the researchers cannot be carried by the researchers personally, at least not when it comes to second and third generations, due to the limitation of time. The researchers themselves are likely to be retired or deceased by the time health issues arise in subsequent generations. Registries for HGE studies have been suggested where information about participants could be stored for extended periods; however, there is still the question of whether the responsibilities and obligations extend over the time span of generations. It should also be noted that there are inherent tensions between protecting the research participants’ health-related data and the need to make the data available for further research.

## Conclusion

For the past few years, the bioethical and biomedical communities have called for an ethically permissible path for HGE to the clinic. While there seems to be ample consensus regarding the necessity for such a pathway, its details remain scarce, and urgent ethical issues that need to be addressed abound. We have highlighted a series of such issues.

It remains a point of contention whether i-HGE can be considered a form of therapy or whether it should be considered a public health measure (to prevent the occurrence of genetic conditions) or a form of reproductive treatment (to help intended parents conceive healthy genetically related offspring). Moreover, it remains an open question whether i-HGE can benefit the offspring, cause harm to a future person, or is neutral with regard to that person. The answers to these questions have consequences when weighing the possible risks associated with i-HGE against its potential. The essential question here remains whether the benefit to the parents of fulfilling their desire to have a genetically related child can outweigh the possibility of harm to the offspring, especially because refraining from these experiments would spare the offspring possible risks. Can it be justified to expose the offspring to the risks of the procedure? If so, why and/or under which conditions?

Being a relatively novel technology, it may prove difficult to conceive of all the risks involved in genome editing, considering that some risks or effects may only manifest years after the application of the technology, at which point they may be difficult or impossible to undo. Not only in light of this translational dilemma should alternatives be considered. What might constitute an alternative to i-HGE remains a subject of debate, and opinions vary on whether there is even a plausible case for i-HGE given the availability of other ARTs, such as PGT.

i-HGE is not exempt from the broader problems that exist in biomedical research including those that concern the quality of preclinical data, on the basis of which progression to clinical trials occurs. Methodological flaws and biases can threaten the scientific validity of preclinical data and of subsequent clinical interventions. Rigorous methodological standards will need to be upheld should HGE ever make its entrance to the clinical sphere.

Whether or when HGE enters clinical practice, participants’ informed consent to the procedure will be an essential requirement. This raises the question of dealing with subsequent non-consent by the offspring and potentially their descendants. Does it constitute an ethical problem in itself that neither offspring nor future generations can consent to the intervention, especially when future generations may be affected by previously unknown or unconsidered long-term effects of the intervention? This also leads to the question of whether and how to organise long-term follow-up studies: to what extent will such studies be required or useful, should participation be made compulsory, and how might long-term follow-up studies affect participants?

Between obtaining consent and conducting long-term follow-up studies, the experimental procedures of i-HGE may be accompanied by significant risks and uncertainties. While risks can be quantified, uncertainties cannot, and they may only manifest some time into the use of a given technology. What needs to be decided is how to approach risks in i-HGE: what level of risk should be deemed acceptable, and how do we reconcile the uneven distribution of risks to the different parties involved (e.g., offspring, intended parents) in i-HGE?

Before any of these issues are given consideration, we must determine for what purpose i-HGE should be undertaken in the first place and which genes would make for suitable targets for i-HGE. It has been suggested that i-HGE should target serious monogenic conditions; however, it is unclear what features make a condition serious, a lack of definition that calls for a viable set of criteria for the seriousness of a disease.

What has become clear is that there remain several important questions that need to be addressed when designing the often-demanded ‘responsible pathway’ for i-HGE to the clinic. Some of these arise from the novelty of the technology and the many uncertainties it entails, others from the fact that i-HGE affects a wider circle of persons than other interventions: the prospective parents, their offspring, and future generations. Regardless of their origins, all these questions must be addressed before clinical translation of i-HGE.

## Footnotes

1 See p. 7 (Article 6, para. 4) of the purported informed consent form for female participants available here: https://web.archive.org/web/20181128061149/http://www.sustc-genome.org.cn/source/pdf/Informed-consent-women-English.pdf.
